# Chromatin marks shape mutation landscape at early stage of cancer progression

**DOI:** 10.1038/s41525-017-0010-y

**Published:** 2017-04-03

**Authors:** Kyungsik Ha, Hong-Gee Kim, Hwajin Lee

**Affiliations:** 1 Biomedical Knowledge Engineering Laboratory, Seoul National University, Seoul, 151-742 South Korea; 2 Interdisciplinary Program of Medical Informatics, College of Medicine, Seoul National University, Seoul, 110-799 South Korea; 3 Dental Research Institute, School of Dentistry, Seoul National University, Seoul, 151-742 South Korea

## Abstract

Somatic mutation rates in cancer differ across the genome in a cancer cell-type specific manner. Although key factors that contribute to the differences were identified, the major cancer progression stage when these factors associate with the mutation variance remained poorly investigated. Here, we analyzed whole-genome sequencing data of pre-cancerous and matching cancer tissues from 173 individuals and 423 normal tissue chromatin features to determine the critical stage of these features contributing to shaping the somatic mutation landscape. Our data showed that the establishment of somatic mutation landscape inferred by chromatin features occur early in the process of cancer progression, and gastric acid reflux environmental exposure-mediated epigenetic changes, represented as gastric metaplasia, at early stage can dramatically impact the somatic mutation landscape. We suggest a possible crucial role of chromatin features during the mutation landscape establishment at early stage of progression in a cancer-type specific manner.

## Cancer: Chromatin marks underlie mutations for early cancer progression

Environmental insults that change how DNA is packaged could lead to mutations responsible for the earliest stages of cancer. Hwajin Lee and colleagues at Seoul National University analyzed whole-genome sequencing data from precancerous tissues (B-cell and esophagus) and the matching cancer types (B-cell leukemia and esophageal cancer) from 173 individuals. They cross checked the mutational profiles against the epigenetic features that shape how DNA is wound into chromosomes, and found that these chromatin marks serve a central role in driving the overall mutation landscape. The authors suggest that gastric acid reflux, which is known to cause a precancerous condition known as Barrett’s esophagus, could be shifting the esophageal cells to become more stomach-like, and the resulting change in chromatin features might be triggering major mutation accumulations that affect subsequent cancer progression.

## Introduction

Recent advances in cancer genomics have so far revealed numerous somatic mutation landscapes for various cancer types, leading to a number of key findings. Identification of new driver gene mutations, deciphering clonal evolution structure, and profiling tumor heterogeneity within and among different patients through examination of mutations, mainly at the gene level,^1–7^ have successfully addressed the genes contributing to cancer progression and identified novel therapeutic targets. Beyond these gene-focused approaches, systematic analyses of mechanisms that could explain genomic regional variations in mutation rates across various cancer types could significantly extend our understanding about common contributors to the establishment of mutation landscapes before and during cancer progression. To this end, a number of studies have examined relationships between regional mutation frequencies across the genome and several types of features, including gene expression level, DNA sequence context, mutation profiles of nucleotide excision and mismatch repair genes, histone post-translational modifications, and open chromatin marks such as DNase1-seq profiles.^8–15^ Although these factors display high correlation with regional mutation rates, somatic mutation profiles used for the studies were limited to fully progressed tumors. Thus, it remains unknown whether the correlations between regional mutation frequencies and cell-of-origin chromatin marks are established either gradually during cancer progression or during a specific critical time period, either pre-or post-malignancy. Analyzing the mutation landscapes of precancerous, non-neoplastic tissues alongside matching cancer tissues could help to determine the major time points where chromatin marks shape the mutation landscape.

Here, we analyzed a total of 38 precancerous lesions including monoclonal B cell lymphocytosis (MBL) and Barrett’s esophagus (BE) (methods). Representative matching cancer types were also analyzed, corresponding to a total of 144 tumor samples from chronic lymphocytic leukemia (CLL) and esophageal adenocarcinoma (EAC). In addition, a total of 14 esophageal squamous cell carcinoma (ESCC) samples were analyzed to represent cancer without any defined precancerous stages during progression with a matching cell-of-origin.

## Results

### Precancerous tissues and matching cancers display similar regional mutation frequency profile

**Fig. 1:**
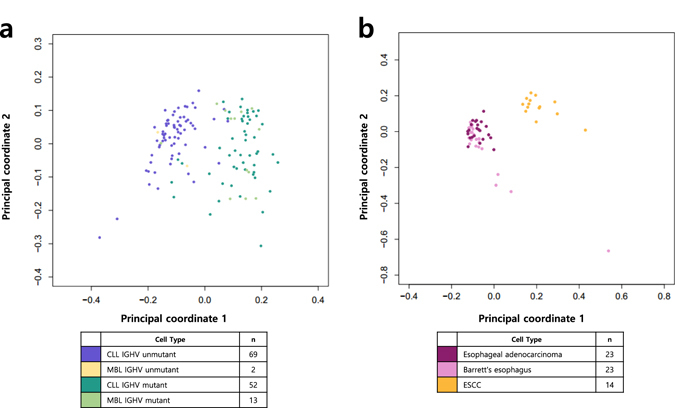
Principal coordinate analysis (PCOA) of individual cancer samples. **a** MBL and CLL with different IGHV mutation status. **b** Barrett’s esophagus, esophageal adenocarcinoma, and ESCC

We first performed principal coordinate analysis (PCOA) to test whether the average mutation rate differences reported previously^16, 17^ were reflected in the level of 1-megabase window regional mutation frequencies. Consistent with the differences in average mutation frequency, both MBL samples and CLL samples were indistinguishably located and formed separate clusters based on immunoglobulin heavy chain variable region (IGHV) mutation status, a key marker for distinguishing either naive-B cells or memory B cell origin for both MBL and CLL.^16, 18^ These results indicate that cell-of-origin differences might contribute to the differences in regional mutation frequencies, rather than cancer progression-based status alone (Fig. 1a). In contrast, individual BE tissues formed clusters with the EAC tissues separate from the ESCC tissues, suggesting that the matching of cancer progression history might serve as a stronger factor than the cell-of-origin context (Fig. 1b). Collectively, these results show similarity in regional variation in mutation frequencies of precancerous tissues and matching cancer types and the differential effect of cell-of-origin context depending on the cancer types.

### IGHV mutation context distinguishes the effect of CD19 chromatin features on the establishment of the somatic mutation landscape for both MBL and CLL

**Fig. 2:**
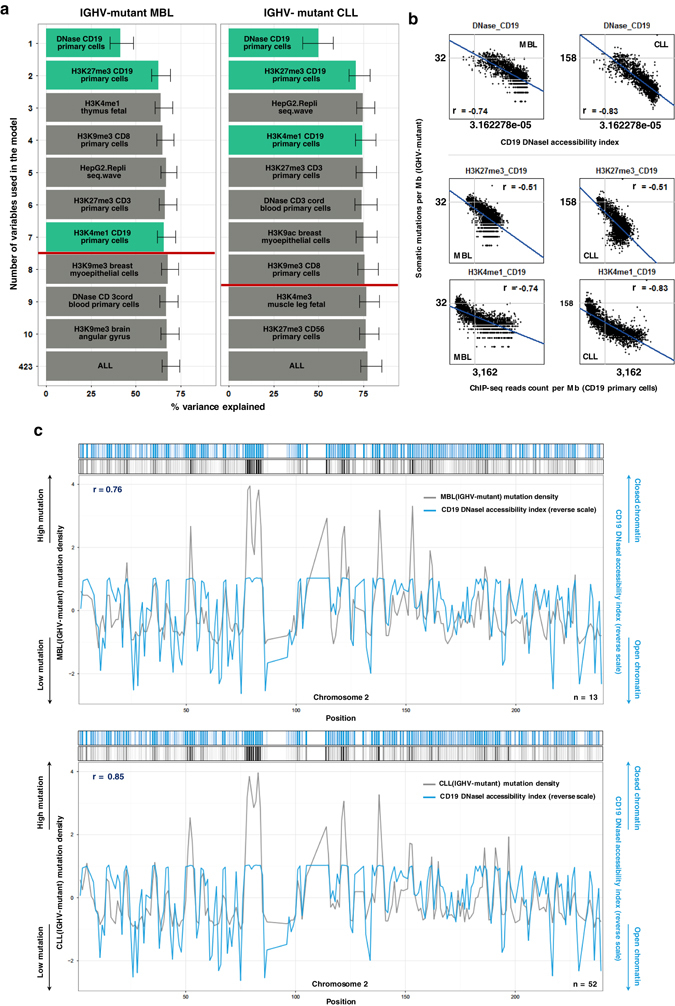
Cell-of origin chromatin features representing association with the regional mutation frequency of CLL and the corresponding precancerous cell type, MBL. **a** Random forest regression-based chromatin feature selection in relations to the regional mutation frequency of IGHV-mutant MBL and CLL samples. Each chromatin feature is ranked by importance value, and variance explained scores are represented by bar length. Error bars demonstrate minimum and maximum values derived from 1000 repeated simulations. *Red lines* display variance explained scores determined by 423 features—1 SEM, and CD19 chromatin features are *green*-colored. **b** Univariate correlation between CD19 chromatin features that displayed significance in the feature selection models and the regional mutation density of IGHV-mutant MBL or CLL. Spearman’s rank correlations (*r*) are shown on each plot. **c** The density plot for regional mutation density of IGHV-mutant MBL or CLL and CD19 DNase1 accessibility index (reverse scale) across the full chromosome 2. Spearman’s rank correlations (*r*) are shown on each plot

Whole-genome analyses of distinct cancer types depict cell-of-origin chromatin marks as the strongest feature explaining the cancer mutation landscape, with a number of proposed mechanisms.^10^ Based on the IGHV mutation status-based clustering of MBL and CLL tissues in PCOA, we hypothesized that differential IGHV mutation status would correlate with distinct chromatin features explaining the regional mutation variation, and similar chromatin features would come up as significant when comparing IGHV mutation type-matching MBL and CLL genomes. To confirm the former part of the hypothesis, we first employed a random forest regression-based chromatin feature selection algorithm to identify significant chromatin features explaining the variance in regional mutation rates for different sample groups. Indeed, significant chromatin features explaining regional mutation variations were different between IGHV mutant and unmutant groups (Supplementary Fig. 1a). Top-ranked chromatin features for both groups were derived from CD19-positive cells, which is expected since the CD19 marker cannot distinguish between naive and memory B cells. To further examine whether the differences in chromatin features were cell-type dependent, we performed chromatin feature selection after removing the 1 Mbp regions containing IGHV mutation status-associated differential DNA methylation single-nucleotide polymorphisms (SNPs), which also highly overlaps with differential DNA methylation SNPs between naive and memory B cells.^19–21^ This approach resulted in 3 out of 4 top significant chromatin features overlapping between the IGHV-mutant and unmutant groups (Supplementary Fig. 1b), implying that the differential chromatin features explaining mutation frequency landscapes of distinct IGHV mutation status might actually correlate with differences in cell-of-origin context. Next, we compared chromatin features that might explain regional mutation variations across the genomes of IGHV-mutation-status-matched MBL and CLL tissues. Due to the limits of sample size and average mutation rate of the samples, only IGHV-mutant MBL and CLL genomes were subjected to further analyses. Notably, the top ranked chromatin feature explaining the mutation variance was identical between IGHV-mutant MBL and CLL samples (Fig. 2a), implicating that the subset of chromatin marks might commonly infer the formation of regional mutation landscape for both pre-cancerous tissues and matching cancer type. Additional examination of simple correlation between regional mutation frequency and histone modification levels derived from CD19-positive cells at the 1-megabase-level revealed no apparent differences between MBL and CLL tissues (Fig. 2b and Supplementary Fig. 2a). The correlation between the CD19 DNase1-seq profile or CD19 H3K4me1 and regional mutation frequency was higher for CLL than MBL for chromosome 2 (Fig. 2c) and other chromosomes (Supplementary Fig. 3a), but this finding might be due to the different number of samples between MBL and CLL, as the correlation score for MBL for all of the chromosome was highly similar to the correlation scores for CLL (−0.76 vs. −0.75 for DNase1-seq, −0.74 vs.−0.75 for H3K4me1) after sample-number matching. These results demonstrate that the cell-of-origin chromatin context, defined by the IGHV mutation status, serves a major role in shaping the mutation landscape of both MBL and CLL tissues, suggesting that the cell-of-origin chromatin landscape could shape the establishment of the somatic mutation landscape of CLL, even before the precancerous cell type, MBL, is apparent.

### Epigenetic shifts caused by metaplasia, driven by acid reflux, explains the establishment of the somatic mutation landscape for both BE and EAC

**Fig. 3:**
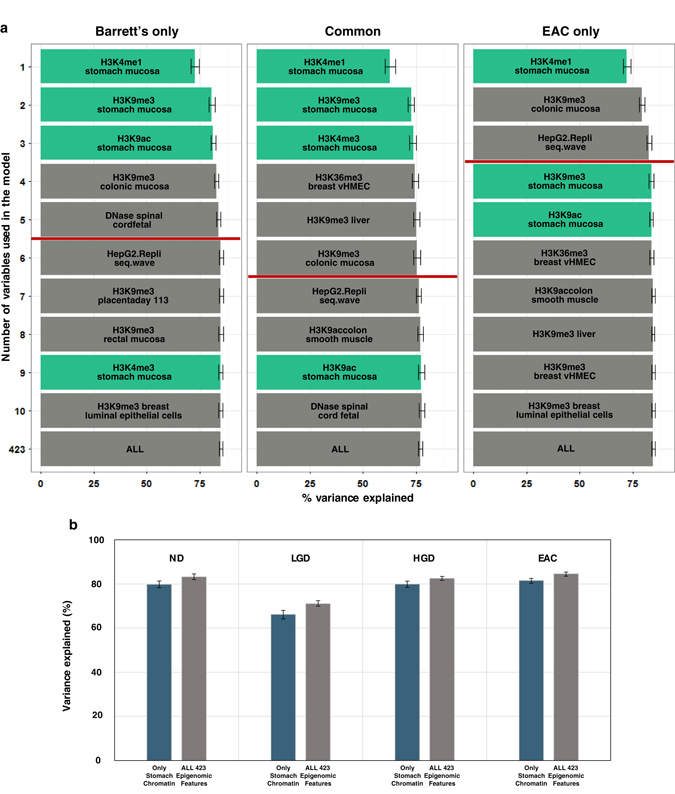
Regional mutation frequency landscape of Barrett’s esophagus and matching esophageal adenocarcinoma are affected by cell-type-shift-associated epigenetic changes. **a** Chromatin feature selection based on the commonality of mutations in paired samples of Barrett’s esophagus and esophageal adenocarcinoma. Barrett’s only: mutations observed only in the Barrett’s esophagus genome, Common: mutations observed in common for both Barrett’s esophagus and esophageal adenocarcinoma genomes, EAC only: mutations observed solely in the esophageal adenocarcinoma genome. **b** Bar graph representing average variance explained scores using either stomach chromatin features (*navy*) or all 423 epigenomic features (*gray*). ND: no dysplasia, LGD: low-grade dysplasia, HGD: high-grade dysplasia, EAC: esophageal adenocarcinoma. Error bars demonstrate minimum and maximum values derived from 1000 repeated simulations

Cell type shift, represented as repeated gastric acid reflux-derived gastric metaplasia, is one of the main hallmarks in the development of BE.^22^ Thus, one could assume that the critical time point for the establishment of the mutation landscape for BE could be either before or during the course of cell type shift, implying the gradual mutation accumulation process, or after its completion, suggesting non-gradual mutation accumulation along the course of cancer progression. Chromatin feature selection analysis of the mutation landscape of BE and EAC tissues confirmed that high-ranked chromatin features were derived from the stomach tissue type, an epigenetically representative matching tissue for gastric metaplasia, for both tissues, without any significant esophageal chromatin features (Supplementary Fig. 4). Simple correlation between regional mutation frequency and histone modification marks from stomach and esophagus tissues revealed no apparent differences between BE and EAC tissues (Supplementary Fig. 2b, c), and this pattern was also consistent with the correlation to stomach tissue DNase1-seq profile (Supplementary Fig. 3b). Moreover, six features covering all stomach chromatin features subjected to the feature selection analysis solely explained over 80% of the regional mutation variance for both BE and EAC tissues, which is unlikely to be random (*p*-value < 2.2e−16) (Supplementary Fig. 5). These results imply that the major time point of mutation landscape establishment for BE is most likely to be after the cell type shift into stomach mucosa-like cells. Chromatin feature selections on subgroups of somatic mutations for BE and EAC based on overlap and uniqueness of the mutations shared common top-ranked stomach chromatin features (Fig. 3a), further supporting the establishment of mutation landscape after the metaplasia. In addition, chromatin feature selection on sample subgroups with respect to dysplasia grades revealed that the top features all originated from stomach tissue (Supplementary Fig. 6) and the variance explained level for all of the dysplasia-based subgroups using six stomach tissue chromatin features were similar to the variance explained level using all 423 chromatin features (Fig. 3b). These findings were consistent with the high correlation to stomach tissue DNase1-seq profile (Supplementary Fig. 3c). Next, we sought to further determine whether the contribution of stomach mucosa chromatin features were indeed more crucial than esophagus chromatin features for shaping the mutation landscape of BE through an independent type of analysis. For this, H3K4me1 chromatin feature was used since this single feature explains most of the variance in mutation frequency of BE. Ninety-two 1-megabase regions displaying differential H3K4me1 levels were selected (methods) based on the speculation that these regions would likely to represent accelerated mutation accumulations through epigenetic changes during gastric metaplasia. Subsequently, we predicted mutation frequencies in the 92 regions by linear regression-based modeling using H3K4me1 level of either stomach mucosa or esophagus tissue (methods). Comparing the observed and predicted mutation frequencies in the 92 regions revealed that the mutation frequencies predicted by H3K4me1 of stomach mucosa was similar to the observed regional mutation frequencies, but the mutation frequencies predicted by H3K4me1 of esophagus tissue was significantly different from the other two groups (Supplementary Fig. 7a). Moreover, regions with larger differences in H3K4me1 level overall display higher accuracy of mutation frequency predicted by using H3K4me1 level of stomach mucosa (Supplementary Fig. 7b). These result further implicate that the chromatin features from stomach mucosa provide major contribution for establishing the mutation landscape of BE, as opposed to the chromatin features of esophagus tissue, a cell-of-origin for BE. From all of these results, we infer an early time point for establishment of the mutation landscape for EAC, even prior to the occurrence of dysplasia for BE, but most likely after epigenetic changes due to gastric metaplasia.

### Cell-of-origin of major chromatin features associated with mutation landscape establishment for BE, EAC, and ESCC are different

**Fig. 4:**
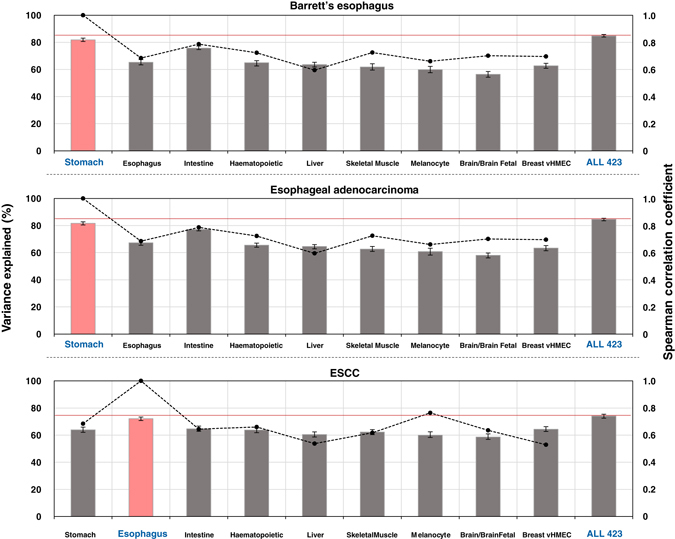
Regional mutation frequency landscape of esophageal squamous cell carcinoma demonstrates the uniqueness of significant chromatin features associated with the Barrett’s esophagus and esophageal adenocarcinoma genomes. Average variance explained scores for pre-cancerous or matching cancer genomes were separately calculated using the tissue or cell type-based subgroup-classified chromatin features. The *pink panel* represents subgroups with the highest variance explained score for each cell type. The *red line* indicates the variance explained score when using all 423 epigenomic features. *Dots* represent the Spearman’s rank correlations (*r*) of chromatin features between the highest variance explained-scored subgroup and the remaining subgroups. Error bars demonstrate minimum and maximum values derived from 1000 repeated simulations

To ensure that the chromatin features shaping the mutation landscape of BE and EAC were not common for any esophageal cancer type, we analyzed the genome of ESCC, another cancer type derived from the esophageal squamous epithelium without any precancerous stages going through cell type shift during the progression. Although the regional mutation frequency of ESCC displayed univariate correlation with histone modification marks from stomach and esophagus tissues in a similar manner (Supplementary Fig. 2d), chromatin feature selection revealed a subset of squamous cell type and esophagus chromatin features that were significant and distinct from BE and EAC (Supplementary Fig. 8). Moreover, measuring the level of variance explained values per tissue or cell type categories showed stomach chromatin features to be the strongest ones for BE and EAC, reaching higher than 90% of the variance level explained by the 423 total chromatin features, whereas esophageal chromatin features were dominant for ESCC (Fig. 4). Notably, the variance explained values for each category displayed non-significant relationship with simple correlations between the chromatin marks from different tissue or cell types (BE *r*_*s*_ = 0.24, EAC *r*_*s*_ = 0.24, ESCC *r*_*s*_ = −0.12). These results imply a distinct process of mutation landscape establishment for these cancer types that varies depending on the presence of precancerous tissues with cell-type shifts.

## Discussion

In this study, we performed an integrative analyses utilizing whole-genome sequencing data from 173 individuals representing precancerous tissues (MBL and BE) and matching cancer types (CLL, BE, and ESCC) combined with 423 epigenomic features derived from normal tissues to elucidate the major time point at which these features play a role in establishing mutation landscape of cancer at early-stage of the progression. These analyses were important to investigate the possible role of chromatin features on shaping cancer somatic mutation landscape at early stage of progression along with possibly inferring previously uncovered intermediate stage of precancerous tissue development in terms of somatic mutation accumulations.

One of the major findings in our work is that the major time point for the establishment of the mutation landscape shaped by chromatin marks could be early, even prior to the phenotypic emergence of precancerous tissues. This finding was consistent for both of the precancerous tissues, MBL, and BE. Although additional whole-genome sequencing data on other types of precancerous tissues might further confirm our findings, these two tissue types do represent two major categories of pre-cancerous tissues, one with metaplasia and one without it.

One thing to note is that our results display non-universal chromatin features identified as significant in different cancer types. The reason for these differences in the extent of variance explaining values for any distinct chromatin feature could be complex, and the reason might be due to the tissue type-dependent differences in the mechanisms of epigenetic regulation plus the differences in major contributing chromatin features serving as either euchromatin or heterochromatin marks. One mechanistic approach to assess the extent of chromatin features contributing to mutation landscape is using CRISPR-Cas9 system to incorporate mutations on chromatin enzymes leading to global epigenetic changes, and then inducing somatic mutations using various types of mutagens to examine the effect of different epigenetic features on shaping mutation landscape, which could be one of the strong candidates for any follow-up research.

Finally, analyses results from BE and EAC also raise the possibility that epigenetic changes due to environmental insults, represented as a cell type shift, could serve as a primary role for establishing the mutation landscape of at early stage of cancer progression. Although there are possibilities that esophagus tissue chromatin features could still be involved in shaping the mutation landscape of BE in a minor manner, our analyses demonstrated that the stomach tissue chromatin features serve as a key factor shaping regional variations in somatic mutation frequency of BE.

Taken together, this study provides novel insights to the mechanisms acting on shaping somatic mutation landscape at early-stage cancer progression, as well as suggesting possible dynamic contributions of chromatin features in this process. Based on our findings, we propose that the mutation accumulation process at early stage of the cancer progression could be non-gradual with possible intermediate stage depending on the presence of metaplasia (Supplementary Fig. 9). Further comprehensive studies to decipher the mutation landscape of other precancerous tissues with metaplasia and discover the exact mechanisms controlling the timing of mutation landscape establishment would lead to a better understanding of the effect of epigenetic marks on shaping the precancerous tissues and matching cancer genome and help identify possible biomarkers for early-stage detection of cancer.

## Methods

### Data

For the purposes of our project, we used somatic mutation data from CLL, MBL, BE, EAC, and ESCC tissues. In the case of CLL and MBL genome data, total mutations were acquired from Supplementary Table 2 of the publication,^16^ consisting of 136 samples (13 IGHV-mutant MBL, 2 IGHV-unmutant MBL, 52 IGHV-mutant CLL, and 69 IGHV-unmutant CLL). In the case of BE, EAC, and ESCC, data use were authorized from ICGC (http://icgc.org) and BGI (http://www.genomics.cn/) before use. A total of 23 pairs of BE and matching EAC genomics data^17^ were authorized from ICGC and genome data of 14 ESCC samples^23^ were acquired from BGI. These data sets were subsequently analyzed following the standard GATK pipeline (https://www.broadinstitute.org/gatk/) and somatic variants were called using the MuTect algorithm^24^ (https://www.broadinstitute.org/cancer/cga/mutect).

A total of 423 epigenomics and chromatin data were from the NIH Roadmap Epigenomics Mapping Consortium^25^ and ENCODE.^26^ NIH Roadmap Epigenomics data were accessible from the NCBI GEO series GSE18927, referring to the University of Washington Human Reference Epigenome Mapping Project.

To calculate the regional mutation density and mean signal of chromatin features, all autosomes were split in 1-Mbp regions followed by filtering out regions containing centromeres, telomeres, and low quality unique mappable base pairs. To determine regional mutation density and histone modification profiles, we counted the total number of somatic mutations or ChIP-seq reads per each 1-megabase region. For analyzing the DNase I hypersensitivity and Repli-seq data, scores of DNase I peaks and replication were calculated per each 1-megabase region. For somatic mutations, ChIP-seq data and DNase I hypersensitivity data, BEDOPS^27^ was employed to calculate the frequency and scores per each 1 Mbp region.

### Principal coordinate analysis

PCOA was used to represent differences in mutation frequency distribution among the individual samples. A dissimilarity matrix was built using 1—Pearson correlation coefficient across all samples. Each sample location was assigned in a two-dimensional space using this matrix.

### Feature selection based on random forest regression

A random forest regression-based feature selection algorithm was performed as described^10^ with modifications. Briefly, the training set for each tree was constructed, followed by using out-of-bag data to estimate the mean squared error. Thus, there was no need to perform additional tests for error evaluation. Out-of-bag data were also used to estimate the importance of each variable. In each out-of-bag case, the values corresponding to each variable were randomly permuted, then tested to each tree. Subtracting the score of the mean squared error between the untouched out-of-bag data cases and the variable-*m*-permuted cases, the raw importance score of variable *m* was measured. By calculating the average score of variable *m* in the entire tree, the rank of importance for each variable was determined. A total of 1000 random forest trees were employed to predict mutation density using a total of 423 chromatin features. Every random forest model was repeated 1000 times.

After the random forest algorithm step, greedy backward elimination was performed to select the top 20 chromatin variables. Subsequent removal of the lowest rank variable was done to calculate the variance explained value measurements for each variable. To conduct feature selection on all of the samples corresponding to the particular pre-cancerous tissues or cancer types, mutation density was calculated by adding samples in each case. However, a number of particular analyses employed the subgrouping of samples. In the case of chromatin feature selection assessing the effects of differential DNA methylation between IGHV-mutants and unmutants (Supplementary Fig. 1b), a total of 935 regions containing differentially methylated CpGs^20^ were removed prior to the analysis. To perform feature selection classified by differential dysplasia states (Supplementary Fig. 6), samples were divided into three groups: 17 samples of no dysplasia, 3 samples of low-grade dysplasia, and 2 samples of high-grade dysplasia. In the case of feature selection after subgrouping for distinct and common mutations (Fig. 3a), all mutations in paired-samples of BE and EAC were divided into three different groups: Barrett’s only, EAC only, and common mutations.

### Analysis of mutation frequency variance explained by chromatin features

To examine the effect of a particular cell-type specific chromatin context on explaining regional variability of mutation density across the genome, chromatin features were subgrouped based on the feature selection algorithm. To study the differences in variance explained values among distinct cell types, nine groups were categorized (Fig. 4). Each group included five chromatin markers common among the groups: H3K27me3, H3K36me3, H3K4me1, H3K4me3, and H3K9me3. Random selection of six chromatin features were either from all of the 423 features or 417 features (excluding stomach mucosa chromatin features) (Supplementary Fig. 5). Random selection of chromatin features was repeated 1000 times, then the average variance explained values and permutation distributions were obtained.

### Prediction of regional mutation frequencies in 1-megabase genomic regions with differential chromatin levels

To select 1-megabase genomic regions with differential H3K4me1 levels, we calculated residual values derived from a linear regression model between the H3K4me1 level of stomach mucosa and that of esophagus tissue. To represent regions harboring differential H3K4me1 levels along with increased mutation accumulation rates after gastric metaplasia, a total of 92 regions were chosen based on the two criteria: (1) displaying top 5% in term of the residual values, (2) showing higher H3K4me1 levels in esophagus than stomach mucosa. Subsequently, we built two separate regression models, and then applied the model to predict the regional mutation frequencies for the 92 regions. One regression model was between observed mutation frequencies in BE with no dysplasia and H3K4me1 level of stomach mucosa, and the other one was between observed mutation frequencies in BE with no dysplasia and H3K4me1 level of esophagus.

### Code availability

Our core analysis code utilizing the random forest feature selection algorithm is available on GitHub (code name: Random_forest_Ha_mutation_epi).

### Preprint availability

The preprint version of our manuscript is currently online at bioarxiv (https://doi.org/10.1101/074724).

## Electronic supplementary material

Supplementary Information
