# Supplementary material for: Chromatin marks shape mutation landscape at early stage of cancer progression
Source: NPJ Genom Med. 2017 Apr 3;2:9. doi: 10.1038/s41525-017-0010-y (PMC5642383; doi:10.1038/s41525-017-0010-y)
Supplement: Supplementary file 1 — Supplementary Information [file 41525_2017_10_MOESM1_ESM.docx]

**Title**

Chromatin marks shape mutation landscape at early stage of cancer progression

**Supplementary Information**

- Supplementary Figure 1 to 9 and Supplementary Table 1

**Supplementary Figures**

**Supplementary Figure 1. Random forest regression-based chromatin feature selection using IGHV-mutant sample groups or IGHV-unmutant sample groups.**

(**a**) IGHV-mutant vs. IGHV-unmutant samples with all 1 megabase genomic regions. (**b**) IGHV-mutant vs. IGHV-unmutant samples without 935 of the 1 megabase genomic regions corresponding to regions containing differentially methylated CpGs between IGHV-mutant and IGHV-unmutant CLL samples.

**Supplementary Figure 2. Correlation plots between regional mutation density and cell-type matching chromatin features.**

(**a**) Mutation density of IGHV-mutant MBL or CLL versus CD19 chromatin features. (**b**) Mutation density of Barrett’s esophagus versus stomach mucosa or esophagus chromatin features. (**c**) Mutation density of esophageal adenocarcinoma versus stomach mucosa or esophagus chromatin features. (**d**) Mutation density of ESCC versus stomach mucosa or esophagus chromatin features.

**Supplementary Figure 3. Spearman’s rank correlation (*r*) between regional mutation density and chromatin accessibility index across the different chromosomes.**

(**a**) MBL and CLL with different IGHV mutation status. (**b**) Barrett’s esophagus, esophageal adenocarcinoma and ESCC. (**c**) Subgroups of Barrett’s esophagus classified by dysplasia states.

**Supplementary Figure 4. Chromatin feature selection in relation to the regional mutation frequency of Barrett’s esophagus and esophageal adenocarcinoma.**

Chromatin features of the stomach mucosa are green-colored.

**Supplementary Figure 5. Comparison of variance explained scores using either stomach chromatin features or groups of randomly selected chromatin features.**

Stomach chromatin group represents a total of 6 chromatin features from stomach tissue. A total of 417 and 423 chromatin groups displayed 6 randomly selected chromatin features from either 417 or 423 features. The difference between 417 and 423 features was the presence or absence of stomach chromatin features. (**a**) Average variance explained scores using 3 different chromatin groups or all of the 423 features. Error bars demonstrate minimum and maximum values derived from 1,000 repeated simulations. (**b**) Distribution of variance explained scores for the group of 6 randomly selected chromatin features from either 417 or 423 chromatin features with 1,000 permutations. Pink-colored distributions represent average variance explained score of stomach chromatin features.

**Supplementary Figure 6. Feature Selection in Barrett’s esophagus and esophageal adenocarcinoma classified by dysplasia status.**

**Supplementary Figure 7. Comparison of observed and predicted mutation frequencies in 1 megabase genomic regions with differential chromatin level.**

(**a**) Boxplot for all 1 megabase genomic regions displaying differential chromatin level (n = 92). Statistical significance was calculated by using Krushal-Wallis one-way ANOVA followed by Dunn’s test (∗∗∗, P < 0.001 ; NS, not significant). (**b**) Heatmap of differences in mutation frequency for the 1 megabase regions with differential chromatin level (n=92). Each square block represents the average mutation frequency differences in 10 regions, except for the 9^th^ square, representing 12 regions. Regions with higher differences in chromatin levels are positioned on the left side of the figure. S-E: predicted number of mutations by H3K4me1 level of stomach mucosa subtracted by predicted number of mutations by H3K4me1level of esophagus tissue, O-E: observed number of mutations in BE with no dysplasia subtracted by predicted number of mutations by H3K4me1level of esophagus tissue, O-S: observed number of mutations in BE with no dysplasia subtracted by predicted number of mutations by H3K4me1level of stomach mucosa.

**Supplementary Figure 8. Chromatin feature selection in relation to the regional mutation frequency of ESCC samples.**

Chromatin features of the esophagus are green-colored**.**

**Supplementary Figure 9. Proposed model showing the major time point for the establishment of the mutation landscape with respect to chromatin features.**

**Supplementary Table 1. List of 423 epigenomic features used in the analyses.**

| H3K27ac adrenal gland | DNase heart fetal |
| --- | --- |
| H3K36me3 adrenal gland | H3K27me3 heart fetal |
| H3K4me1 adrenal gland | H3K36me3 heart fetal |
| H3K9me3 adrenal gland | H3K4me1 heart fetal |
| DNase adrenal gland fetal | H3K4me3 heart fetal |
| H3K27ac adrenal gland fetal | H3K9ac heart fetal |
| H3K27me3 adrenal gland fetal | H3K9me3 heart fetal |
| H3K36me3 adrenal gland fetal | DNase IMR90 cell line |
| H3K4me1 adrenal gland fetal | H3K27ac IMR90 cell line |
| H3K4me3 adrenal gland fetal | H3K27me3 IMR90 cell line |
| H3K9me3 adrenal gland fetal | H3K36me3 IMR90 cell line |
| H3K27ac bladder | H3K4me1 IMR90 cell line |
| H3K36me3 bladder | H3K4me3 IMR90 cell line |
| H3K4me1 bladder | H3K9ac IMR90 cell line |
| H3K27ac brain angular gyrus | H3K9me3 IMR90 cell line |
| H3K27me3 brain angular gyrus | DNase iPS DF 19.11 cell line |
| H3K36me3 brain angular gyrus | H3K27ac iPS DF 19.11 cell line |
| H3K4me1 brain angular gyrus | H3K27me3 iPS DF 19.11 cell line |
| H3K4me3 brain angular gyrus | H3K36me3 iPS DF 19.11 cell line |
| H3K9ac brain angular gyrus | H3K4me1 iPS DF 19.11 cell line |
| H3K9me3 brain angular gyrus | H3K4me3 iPS DF 19.11 cell line |
| H3K27ac brain anterior caudate | H3K9me3 iPS DF 19.11 cell line |
| H3K27me3 brain anterior caudate | DNase iPS DF 19.7 cell line |
| H3K36me3 brain anterior caudate | DNase iPS DF 4.7 cell line |
| H3K4me1 brain anterior caudate | DNase iPS DF 6.9 cell line |
| H3K4me3 brain anterior caudate | H3K27ac iPS DF 6.9 cell line |
| H3K9ac brain anterior caudate | H3K27me3 iPS DF 6.9 cell line |
| H3K9me3 brain anterior caudate | H3K36me3 iPS DF 6.9 cell line |
| H3K27ac brain cingulate gyrus | H3K4me1 iPS DF 6.9 cell line |
| H3K27me3 brain cingulate gyrus | H3K4me3 iPS DF 6.9 cell line |
| H3K36me3 brain cingulate gyrus | H3K9me3 iPS DF 6.9 cell line |
| H3K4me1 brain cingulate gyrus | H3K36me3 kidney |
| H3K4me3 brain cingulate gyrus | H3K4me1 kidney |
| H3K9ac brain cingulate gyrus | H3K4me3 kidney |
| H3K9me3 brain cingulate gyrus | H3K9ac kidney |
| H3K27me3 brain dorsal neocortex fetal | H3K9me3 kidney |
| H3K4me3 brain dorsal neocortex fetal | DNase kidney fetal |
| H3K9ac brain dorsal neocortex fetal | H3K27me3 kidney fetal |
| DNase brain fetal | H3K36me3 kidney fetal |
| H3K27me3 brain fetal | H3K4me1 kidney fetal |
| H3K36me3 brain fetal | H3K4me3 kidney fetal |
| H3K4me1 brain fetal | H3K9ac kidney fetal |
| H3K4me3 brain fetal | H3K9me3 kidney fetal |
| H3K9me3 brain fetal | DNase kidney left fetal |
| H3K27me3 brain germinal matrix fetal | DNase kidney renal cortex fetal |
| H3K36me3 brain germinal matrix fetal | DNase kidney renal cortex left fetal |
| H3K4me1 brain germinal matrix fetal | DNase kidney renal cortex right fetal |
| H3K4me3 brain germinal matrix fetal | DNase kidney renal pelvis fetal |
| H3K9me3 brain germinal matrix fetal | DNase kidney renal pelvis left fetal |
| H3K27ac brain hippocampus middle | DNase kidney renal pelvis right fetal |
| H3K27me3 brain hippocampus middle | DNase kidney right fetal |
| H3K36me3 brain hippocampus middle | DNase large intestine fetal |
| H3K4me1 brain hippocampus middle | H3K27ac large intestine fetal |
| H3K4me3 brain hippocampus middle | H3K27me3 large intestine fetal |
| H3K9ac brain hippocampus middle | H3K36me3 large intestine fetal |
| H3K9me3 brain hippocampus middle | H3K4me1 large intestine fetal |
| H3K27ac brain mid frontal Brodmann  area 9/46 dorsolateral prefrontal cortex | H3K4me3 large intestine fetal |
| H3K27me3 brain mid frontal Brodmann  area 9/46 dorsolateral prefrontal cortex | H3K9me3 large intestine fetal |
| H3K36me3 brain mid frontal Brodmann  area 9/46 dorsolateral prefrontal cortex | H3K27me3 liver |
| H3K4me1 brain mid frontal Brodmann  area 9/46 dorsolateral prefrontal cortex | H3K36me3 liver |
| H3K4me3 brain mid frontal Brodmann  area 9/46 dorsolateral prefrontal cortex | H3K4me1 liver |
| H3K9ac brain mid frontal Brodmann  area 9/46 dorsolateral prefrontal cortex | H3K4me3 liver |
| H3K9me3 brain mid frontal Brodmann  area 9/46 dorsolateral prefrontal cortex | H3K9ac liver |
| H3K27me3 breast luminal epithelial cells | H3K9me3 liver |
| H3K36me3 breast luminal epithelial cells | H3K27ac lung |
| H3K4me1 breast luminal epithelial cells | H3K36me3 lung |
| H3K9me3 breast luminal epithelial cells | H3K4me1 lung |
| H3K27me3 breast myoepithelial cells | H3K9me3 lung |
| H3K36me3 breast myoepithelial cells | DNase lung fetal |
| H3K4me1 breast myoepithelial cells | H3K27me3 lung fetal |
| H3K4me3 breast myoepithelial cells | H3K36me3 lung fetal |
| H3K9ac breast myoepithelial cells | H3K4me1 lung fetal |
| H3K9me3 breast myoepithelial cells | H3K4me3 lung fetal |
| DNase breast vHMEC | H3K9ac lung fetal |
| H3K27me3 breast vHMEC | H3K9me3 lung fetal |
| H3K36me3 breast vHMEC | DNase lung left fetal |
| H3K4me1 breast vHMEC | DNase lung right fetal |
| H3K4me3 breast vHMEC | DNase muscle arm fetal |
| H3K9me3 breast vHMEC | DNase muscle back fetal |
| DNase CD14 primary cells | DNase muscle leg fetal |
| H3K27ac CD14 primary cells | H3K27ac muscle leg fetal |
| H3K27me3 CD14 primary cells | H3K27me3 muscle leg fetal |
| H3K36me3 CD14 primary cells | H3K36me3 muscle leg fetal |
| H3K4me1 CD14 primary cells | H3K4me1 muscle leg fetal |
| H3K4me3 CD14 primary cells | H3K4me3 muscle leg fetal |
| H3K9me3 CD14 primary cells | H3K9me3 muscle leg fetal |
| DNase CD19 primary cells | DNase muscle lower limb fetal |
| H3K27ac CD19 primary cells | DNase muscle trunk fetal |
| H3K27me3 CD19 primary cells | H3K27ac muscle trunk fetal |
| H3K36me3 CD19 primary cells | H3K27me3 muscle trunk fetal |
| H3K4me1 CD19 primary cells | H3K36me3 muscle trunk fetal |
| H3K4me3 CD19 primary cells | H3K4me1 muscle trunk fetal |
| H3K9me3 CD19 primary cells | H3K4me3 muscle trunk fetal |
| DNase CD20 primary cells | H3K9me3 muscle trunk fetal |
| DNase CD3 cord blood primary cells | DNase muscle upper back fetal |
| DNase CD3 mobilized primary cells | DNase muscle upper limb fetal |
| DNase CD3 primary cells | DNase muscle upper trunk fetal |
| H3K27ac CD3 primary cells | H3K27ac ovary |
| H3K27me3 CD3 primary cells | H3K36me3 ovary |
| H3K36me3 CD3 primary cells | H3K4me1 ovary |
| H3K4me1 CD3 primary cells | H3K9me3 ovary |
| H3K4me3 CD3 primary cells | DNase ovary fetal |
| H3K9me3 CD3 primary cells | H3K27ac pancreas |
| DNase CD34 mobilized primary cells | H3K27me3 pancreas |
| H3K27ac CD34 mobilized primary cells | H3K36me3 pancreas |
| H3K27me3 CD34 mobilized primary cells | H3K4me1 pancreas |
| H3K36me3 CD34 mobilized primary cells | H3K4me3 pancreas |
| H3K4me1 CD34 mobilized primary cells | H3K9me3 pancreas |
| H3K4me3 CD34 mobilized primary cells | DNase penis foreskin fibroblast primary cells |
| H3K9me3 CD34 mobilized primary cells | H3K27ac penis foreskin fibroblast primary cells |
| DNase CD34 primary cells | H3K27me3 penis foreskin fibroblast primary cells |
| H3K27me3 CD34 primary cells | H3K36me3 penis foreskin fibroblast primary cells |
| H3K36me3 CD34 primary cells | H3K4me1 penis foreskin fibroblast primary cells |
| H3K4me1 CD34 primary cells | H3K4me3 penis foreskin fibroblast primary cells |
| H3K4me3 CD34 primary cells | H3K9me3 penis foreskin fibroblast primary cells |
| H3K9me3 CD34 primary cells | DNase penis foreskin keratinocyte primary cells |
| DNase CD4 mobilized primary cells | H3K27ac penis foreskin keratinocyte primary cells |
| DNase CD4 primary cells | H3K27me3 penis foreskin keratinocyte primary cells |
| H3K36me3 CD4 primary cells | H3K36me3 penis foreskin keratinocyte primary cells |
| H3K4me3 CD4 primary cells | H3K4me1 penis foreskin keratinocyte primary cells |
| DNase CD56 mobilized primary cells | H3K4me3 penis foreskin keratinocyte primary cells |
| DNase CD56 primary cells | H3K9ac penis foreskin keratinocyte primary cells |
| H3K27ac CD56 primary cells | H3K9me3 penis foreskin keratinocyte primary cells |
| H3K27me3 CD56 primary cells | DNase penis foreskin melanocyte primary cells |
| H3K36me3 CD56 primary cells | H3K27ac penis foreskin melanocyte primary cells |
| H3K4me1 CD56 primary cells | H3K27me3 penis foreskin melanocyte primary cells |
| H3K4me3 CD56 primary cells | H3K36me3 penis foreskin melanocyte primary cells |
| DNase CD8 mobilized primary cells | H3K4me1 penis foreskin melanocyte primary cells |
| DNase CD8 primary cells | H3K4me3 penis foreskin melanocyte primary cells |
| H3K27ac CD8 primary cells | H3K9me3 penis foreskin melanocyte primary cells |
| H3K36me3 CD8 primary cells | DNase placenta day105 |
| H3K4me3 CD8 primary cells | DNase placenta day108 |
| H3K9me3 CD8 primary cells | DNase placenta day113 |
| H3K27me3 colon smooth muscle | H3K27me3 placenta day113 |
| H3K36me3 colon smooth muscle | H3K4me3 placenta day113 |
| H3K4me1 colon smooth muscle | H3K9me3 placenta day113 |
| H3K4me3 colon smooth muscle | DNase placenta day85 |
| H3K9ac colon smooth muscle | DNase placenta day91 |
| H3K9me3 colon smooth muscle | H3K27ac psoas muscle |
| H3K27me3 colonic mucosa | H3K27me3 psoas muscle |
| H3K36me3 colonic mucosa | H3K36me3 psoas muscle |
| H3K4me1 colonic mucosa | H3K4me1 psoas muscle |
| H3K4me3 colonic mucosa | H3K9me3 psoas muscle |
| H3K9ac colonic mucosa | H3K27me3 rectal mucosa |
| H3K9me3 colonic mucosa | H3K36me3 rectal mucosa |
| H3K27me3 duodenum mucosa | H3K4me1 rectal mucosa |
| H3K36me3 duodenum mucosa | H3K4me3 rectal mucosa |
| H3K4me1 duodenum mucosa | H3K9ac rectal mucosa |
| H3K4me3 duodenum mucosa | H3K9me3 rectal mucosa |
| H3K9ac duodenum mucosa | H3K27ac sigmoid colon |
| H3K9me3 duodenum mucosa | H3K27me3 sigmoid colon |
| H3K27ac esophagus | H3K36me3 sigmoid colon |
| H3K27me3 esophagus | H3K4me1 sigmoid colon |
| H3K36me3 esophagus | H3K9me3 sigmoid colon |
| H3K4me1 esophagus | H3K27ac skeletal muscle |
| H3K4me3 esophagus | H3K27me3 skeletal muscle |
| H3K9me3 esophagus | H3K36me3 skeletal muscle |
| H3K27ac gastric | H3K4me1 skeletal muscle |
| H3K27me3 gastric | H3K4me3 skeletal muscle |
| H3K36me3 gastric | H3K9ac skeletal muscle |
| H3K4me1 gastric | H3K9me3 skeletal muscle |
| H3K4me3 gastric | DNase skin abdomen fetal |
| H3K9me3 gastric | DNase skin back fetal |
| DNase H1 BMP4 derived  mesendoderm cultured cells | DNase skin biceps left fetal |
| H3K27ac H1 BMP4 derived  mesendoderm cultured cells | DNase skin biceps right fetal |
| H3K27me3 H1 BMP4 derived  mesendoderm cultured cells | DNase skin fetal |
| H3K36me3 H1 BMP4 derived  mesendoderm cultured cells | DNase skin quadricips left fetal |
| H3K4me1 H1 BMP4 derived  mesendoderm cultured cells | DNase skin quadricips right fetal |
| H3K4me3 H1 BMP4 derived  mesendoderm cultured cells | DNase skin scalp fetal |
| H3K9ac H1 BMP4 derived  mesendoderm cultured cells | DNase skin upper back fetal |
| DNase H1 BMP4 derived  trophoblast cultured cells | H3K27ac small intestine |
| H3K27ac H1 BMP4 derived  trophoblast cultured cells | H3K36me3 small intestine |
| H3K27me3 H1 BMP4 derived  trophoblast cultured cells | H3K4me1 small intestine |
| H3K36me3 H1 BMP4 derived  trophoblast cultured cells | H3K4me3 small intestine |
| H3K4me1 H1 BMP4 derived  trophoblast cultured cells | H3K9me3 small intestine |
| H3K4me3 H1 BMP4 derived  trophoblast cultured cells | DNase small intestine fetal |
| H3K9ac H1 BMP4 derived  trophoblast cultured cells | H3K27ac small intestine fetal |
| H3K9me3 H1 BMP4 derived  trophoblast cultured cells | H3K27me3 small intestine fetal |
| DNase H1 cell line | H3K36me3 small intestine fetal |
| H3K27ac H1 cell line | H3K4me1 small intestine fetal |
| H3K27me3 H1 cell line | H3K4me3 small intestine fetal |
| H3K36me3 H1 cell line | H3K9me3 small intestine fetal |
| H3K4me1 H1 cell line | DNase spinal cord fetal |
| H3K4me3 H1 cell line | DNase spleen fetal |
| H3K9ac H1 cell line | DNase stomach fetal |
| H3K9me3 H1 cell line | H3K27ac stomach fetal |
| DNase H1 derived  mesenchymal stem cells | H3K27me3 stomach fetal |
| H3K27ac H1 derived  mesenchymal stem cells | H3K36me3 stomach fetal |
| H3K27me3 H1 derived  mesenchymal stem cells | H3K4me1 stomach fetal |
| H3K36me3 H1 derived  mesenchymal stem cells | H3K4me3 stomach fetal |
| H3K4me1 H1 derived  mesenchymal stem cells | H3K9me3 stomach fetal |
| H3K4me3 H1 derived  mesenchymal stem cells | H3K27me3 stomach mucosa |
| H3K9ac H1 derived  mesenchymal stem cells | H3K36me3 stomach mucosa |
| H3K9me3 H1 derived  mesenchymal stem cells | H3K4me1 stomach mucosa |
| DNase H1 derived neuronal  progenitor cultured cells | H3K4me3 stomach mucosa |
| H3K27ac H1 derived neuronal  progenitor cultured cells | H3K9ac stomach mucosa |
| H3K27me3 H1 derived neuronal  progenitor cultured cells | H3K9me3 stomach mucosa |
| H3K36me3 H1 derived neuronal  progenitor cultured cells | DNase testes fetal |
| H3K4me1 H1 derived neuronal  progenitor cultured cells | H3K27ac thymus |
| H3K4me3 H1 derived neuronal  progenitor cultured cells | H3K36me3 thymus |
| H3K9ac H1 derived neuronal  progenitor cultured cells | H3K4me1 thymus |
| H3K9me3 H1 derived neuronal  progenitor cultured cells | H3K9me3 thymus |
| DNase H9 cell line | DNase thymus fetal |
| H3K27ac H9 cell line | H3K27ac thymus fetal |
| H3K27me3 H9 cell line | H3K27me3 thymus fetal |
| H3K36me3 H9 cell line | H3K4me1 thymus fetal |
| H3K4me1 H9 cell line | H3K4me3 thymus fetal |
| H3K4me3 H9 cell line | H3K9me3 thymus fetal |
| H3K9ac H9 cell line | HepG2.Repli seq.wave |
| H3K9me3 H9 cell line | IMR90.Repli seq.wave |
| DNase heart | Mcf7.Repli seq.wave |
|  | Nhek.Repli seq.wave |
